# *De Novo* Genome Assembly for an Endangered Lemur Using Portable Nanopore Sequencing in Rural Madagascar

**DOI:** 10.1002/ece3.70734

**Published:** 2025-01-06

**Authors:** Lindsey Hauff, Noa Elosmie Rasoanaivo, Andriamahery Razafindrakoto, Hajanirina Ravelonjanahary, Patricia C. Wright, Rindra Rakotoarivony, Christina M. Bergey

**Affiliations:** ^1^ Department of Ecology, Evolution, and Natural Resources Rutgers University New Brunswick New Jersey USA; ^2^ Center for Human Evolutionary Studies Rutgers University New Brunswick New Jersey USA; ^3^ Human Genetics Institute of New Jersey Piscataway New Jersey USA; ^4^ Department of Zoology and Animal Biodiversity University of Antananarivo Antananarivo Madagascar; ^5^ Centre ValBio, Ranomafana National Park Ifanadiana Madagascar; ^6^ Department of Anthropology Stony Brook University Stony Brook New York USA; ^7^ Department of Biological Anthropology and Sustainable Development University of Antananarivo Antananarivo Madagascar; ^8^ Department of Genetics Rutgers University Piscataway New Jersey USA

**Keywords:** Centre ValBio Research Station, conservation genomics, genome assembly, Oxford Nanopore sequencing, red‐fronted brown lemur, reference genome

## Abstract

As one of the most threatened mammalian taxa, lemurs of Madagascar are facing unprecedented anthropogenic pressures. To address conservation imperatives such as this, researchers have increasingly relied on conservation genomics to identify populations of particular concern. However, many of these genomic approaches necessitate high‐quality genomes. While the advent of next‐generation sequencing technologies and the resulting reduction in associated costs have led to the proliferation of genomic data and high‐quality reference genomes, global discrepancies in genomic sequencing capabilities often result in biological samples from biodiverse host countries being exported to facilities in the Global North, creating inequalities in access and training within genomic research. Here, we present the first published reference genome for the endangered red‐fronted brown lemur ( *Eulemur rufifrons* ) from sequencing efforts conducted entirely within the host country using portable Oxford Nanopore sequencing. Using an archived *E. rufifrons* specimen, we conducted long‐read, nanopore sequencing at the Centre ValBio Research Station near Ranomafana National Park, in rural Madagascar, generating over 750 Gb of sequencing data from 10 MinION flow cells. Exclusively using this long‐read data, we assembled 2.157 gigabase, 2980‐contig nuclear assembly with an N50 of 101.6 Mb and a 17,108 bp mitogenome. The nuclear assembly had 30× average coverage and was comparable in completeness to other primate reference genomes, with a 96.1% BUSCO completeness score for primate‐specific genes. As the first published reference genome for *E. rufifrons* and the only annotated genome available for the speciose *Eulemur* genus, this resource will prove vital for conservation genomic studies while our efforts exhibit the potential of this protocol to address research inequalities and build genomic capacity.

## Graphical abstract

Lemurs in Madagascar are under severe threat from human activities, and while advancements in genomic sequencing offer promises for conservation, disparities in global sequencing capabilities often lead to samples being sent to the Global North, perpetuating inequalities in research access and training. We present the first reference genome for the endangered red‐fronted brown lemur from sequencing efforts conducted entirely within the host country using portable nanopore sequencing. Our efforts exhibit the potential of this technology to address research inequalities and build genomic capacity in high‐biodiversity countries.

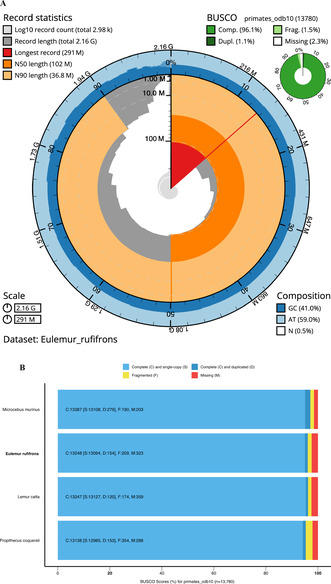

## TENY MALAGASY ABSTRACT

Amin'ny maha‐iray amin’ireo vondrona mampinono atahorana ho lany tamingana indrindra, ny varika eto Madagasikara dia miatrika tsindry avy amin’ny olombelona izay mbola tsy nisy toy izany. Mba hamenoana ireo fepetra momba ny fiarovana ny amin’ny toy izany, dia nitodika betsaka kokoa amin’ny fiarovana mifantoka amin’ny fototarazo ny mpikaroka mba hamantarana ireo vondrona voakasika manokana. Na izany aza, maro amin’ireo fomba fiasa ireo no mila fototarazo avo lenta. Raha ny fahatongavan’ny teknolojia vaovao momba ny famakiana ny fototarazo sy ny fampihenana ny fandaniana mifandraika amin’izany dia nitarika ny fitomboan’ny angon‐drakitra momba ny fototarazo sy ny fototarazo fanoharana avo lenta, ny tsy fitovizan’izao tontolo izao amin’ny fahafahana manao izany famakiana ny fototarazo izany izay matetika miteraka ny fanondranana ireo santionany biolojika avy amin’ireo firenena mpampiantrano manakarena zavaboary makany amin’ireo firenena tavaratra efa mandroso, izay miteraka tsy fitoviana eo amin’ny fahazoana alalana sy ny fiofanana amin’ny fikarohana momba ny fototarazo. Eto izahay dia manolotra ny fototarazo fanoharana voalohany ho an’ny varika mavo (*Eulemur rufifrons*), vondrona atahorana ho lany tamingana, avy amin’ny ezaka famakiana fototarazo izay natao tao amin’ilay firenena mpampiantrano tamin’ny alàlan’ny fitaovana famakiana fototarazo Oxford Nanopore MinION. Tamin’ny alalan’ny santionana *E. rufifrons* voatahiry, dia nanao famakiana lava tamin’ny alalan’ny fitaovana nanopore tao amin’ny tobim‐pikarohana Centre ValBio akaikin’ny valan‐javaboary nasionalin’i Ranomafana, ambanivohitr’i Madagasikara izahay, ka namokatra angon‐drakitra 750 Gb mahery avy amin’ny fitaovana “MinION flow cell” folo. Avy tamin’ny fampiasana manokana an’io angon‐drakitra io, dia nahazo angona nokleary mirefy 2.157 gigabase, miaraka amin’ny contig miisa 2,980, manana N50 mirefy 101.6 Mb, ary angona mitokondria mirefy 17,108 bp. Ilay angona nokleary dia manana taha eo amin’ny 30x eo izay azo ampitahaina amin’ny fahafenoan’ny fototarazo fanoharana primaty hafa ary nahazoana taha‐pahafenoana BUSCO 96.1% ho an’ny singam‐pototarazo manokanan’ny primaty. Amin’ny maha‐fototarazo fanoharana voalohany navoaka ho an’ny *E. rufifrons* ary fanoharana tokana misy ho an’ireo vondrona *Eulemur*, ity angona ity dia manaporofo fa tena ilaina ny fikarohana momba ny fiarovana miopmana amin’ny fototarazo raha toa kosa ny ezaka ataonay dia mampiseho ny mety ho fahafahan’ity drafitrasa ity mamahana ny tsy fitoviana amin’ny lafiny fikarohana sy hanamafisana ny fahaiza‐manao momba ny fototarazo.

## 1 Introduction

Madagascar's endemic lemurs are one of most threatened mammalian groups in the world with 98% of recognized species considered threatened with extinction (IUCN 2023; Schwitzer et al. 2014). Multiple anthropogenic forces have resulted in rapid and severe environmental change on this island nation in the last decades, including significant reductions in forest cover (Vieilledent et al. 2018) on which many of these vulnerable primate species are dependent. Factoring in intensification of climatic events, such as drastic changes in precipitation and amplification of storm severity and frequency, Madagascar's fragmented forests and the lemur populations residing within them face critical challenges (Weiskopf et al. 2021).

To address biodiversity imperatives such as this, genomic approaches have become crucial tools in aiding conservation management decisions by identifying populations of particular conservation value (Hohenlohe, Funk, and Rajora 2021; Supple and Shapiro 2018). Offering increased precision of estimates compared to analyses that use limited sets of genetic markers (Allendorf, Hohenlohe, and Luikart 2010; Hohenlohe, Funk, and Rajora 2021), genome‐based analyses are of particular importance to conservation as they can robustly assess past and present demographic parameters (Abascal et al. 2016; Palkopoulou et al. 2015), adaptive potential (Harrisson et al. 2014; Maier et al. 2022), and extinction risk (Robinson et al. 2022). A limitation to widespread implementation of conservation genomics is the reliance on high‐quality reference genomes (Rhie et al. 2021), which can be costly, laborious, and computationally demanding to produce for non‐model organisms of conservation concern. However, the “genomics revolution” brought upon by the advent of next‐generation, high‐throughput sequencing technologies has resulted in a reduction in costs associated with sequencing and an increase in bioinformatic tools available to handle the considerable amount of data generated by such advances (Schiebelhut et al. 2024).

These developments, in conjunction with growing genome consortia initiatives (Darwin Tree of Life Project Consortium 2022; Koepfli et al. 2015; Lewin et al. 2022), have led to the proliferation of publicly available reference genomes (Formenti et al. 2022). While genomic resources for IUCN Red List species have grown more than threefold in a span of 3 years (Brandies et al. 2019; Hogg et al. 2022), there remains global disparities in access to genomic research with sequencing facilities predominantly located in the Global North (Helmy, Awad, and Mosa 2016). This disproportionate distribution of sequencing centers often results in biological materials from biodiverse regions exported and researchers from host countries excluded from genomic work, creating inequalities in participation and training opportunities within genomic research (Hoban et al. 2013; Shafer et al. 2014). However, portable DNA sequencers, like the MinION from Oxford Nanopore Technologies (ONT), have the potential to help mitigate such discrepancies by facilitating genomic work in developing countries (Kigen et al. 2023; Onwuamah et al. 2021; Rivière et al. 2021), reducing barriers to entry by allowing training and participation on associated genomic protocols while fostering more equitable international partnerships.

Portable MinION sequencing has been employed in remote field conditions in Madagascar to great success. In addition to being able to conduct rapid, in situ biodiversity assessments to confirm the presence of vulnerable Danfoss' mouse lemur ( *Microcebus danfossi* ), one research team was able to use their mobile genetics laboratories to provide a collaborative molecular workshop for local Malagasy students, who previously lacked of access to equipment and hands‐on training as major barriers to pursuing such research (Blanco et al. 2020). Usage of portable ONT sequencing has also produced nanopore‐only reference assemblies (e.g., Flack et al. 2023; Pozo et al. 2024), highlighting its potential to generate valuable genomic resources while providing underrepresented researchers pivotal access and training to molecular techniques.

Here, we present the first published high‐quality reference genome of the red‐fronted brown lemur ( *Eulemur rufifrons* ), assembled exclusively from long‐read sequencing efforts conducted within Madagascar. Red‐fronted brown lemurs are members of the “true lemur” genus (*Eulemur* spp.), one of the most speciose of all of Madagascar's lemurs, with species distributed in remaining forest fragments across the island (Markolf and Kappeler 2013). *E. rufifrons* are distributed in both the eastern rainforests and the western dry forests and live in multi‐male/multi‐female groups (Overdorff et al. 1999). While being primarily frugivorous, *E. rufifrons* . are medium‐sized, arboreal primates that display notable dietary flexibility (Johnson 2007; Overdorff 1993). *Eulemur* spp. are often considered more resilient to anthropogenic disturbances than larger‐bodied lemurs, perhaps due to behavioral plasticity permitting shifts to cathemeral activity in the presence of higher human disturbance (Donati et al. 2016). Despite this perceived hardiness, *Eulemur* spp. are continuing to dwindle, with all 12 species within the genus listed as at least “Vulnerable” to extinction and declining (IUCN 2023). Notably, eastern populations of *E. rufifrons* have undergone significant demographic declines in the last few decades, believed to be associated with decreasing fruit availability (Erhart and Overdorff 2008). Conservation of these species are critical, as the health and biodiversity of Madagascar's tropical forests are intricately woven to the persistence of diverse, frugivorous lemur communities, with many plant species relying nearly exclusively on a specific lemur taxon for seed dispersal and germination (Wright et al. 2011). As at the time of publication there are no other publicly available annotated reference genomes for the *Eulemur* genus. We anticipate our work will be a valuable genomic resource for conservation efforts across Madagascar as well as a proof of concept for how high‐quality genome assemblies can be generated for such species of conservation concern.

## 2 Materials and Methods

### 2.1 DNA Sample and Sequencing

#### 2.1.1 DNA Sample

To conduct whole‐genome sequencing within Madagascar, we extracted DNA from tissue of an adult female *Eulemur rufifrons* archival specimen that was opportunistically collected within Ranomafana National Park (date unknown) and stored whole at −20°C at the nearby Centre ValBio Research Station. As all sequencing efforts were performed over the course of two time periods, with the pilot study conducted in August 2022 and the bulk of the sequencing conducted in July 2023, the samples and protocols used were adapted over time to account for protocol improvements and unanticipated challenges associated with conducting genomic work in remote regions, as noted below. Skin (totaling 20 mg) was sampled in 2022 and used as the original tissue sample for genomic DNA extractions with Monarch High Molecular Weight (HMW) Extraction Kit for Tissue (New England Biolabs). Spleen tissue (totaling 25 mg) was sampled in 2023 and used for extractions with the DNeasy Blood and Tissue kit (Qiagen) as a comprehensive dissection of the specimen occurred between sampling endeavors and the HMW kit was found to be no longer viable, perhaps due to storage conditions in the rainforest. For all extractions conducted, DNA quantity and quality were assessed via a spectrophotometer, NanoDrop 2000 (Thermo Scientific).

#### 2.1.2 Library Preparation

During the pilot stage of sequencing for this project, 2 μg of HMW DNA was used as input across two library preparations using the Field Sequencing Kit (Oxford Nanopore SQK‐LRK001) and following the manufacturer's protocol. During the second phase of the project, 6 μg of the remaining HMW DNA and 7 μg of the spleen‐extracted DNA were used as input for library preparations with the Oxford Nanopore Ligation Sequencing kit SQK‐LSK109, split into three and two library preparations for each extraction, respectively (Table S1). Modifications as described by Flack et al. (2023) were implemented during the latter sequencing effort to scale up the library preparation reaction and input more DNA to generate additional library material for sequencing, allowing for multiple reloadings of the same library and flow cell between washes. Prepared libraries were stored at 4°C while awaiting sequencing.

#### 2.1.3 Sequencing

All genomic sequencing was conducted within Madagascar at the Centre ValBio Research Station on portable MinION sequencers (Mk1C and Mk1B; Oxford Nanopore Technologies). To operate the MinION Mk1B sequencer, an Alienware ×17 R2 laptop was used with the following specifications: Windows 11 Pro operating system, Intel Core i7 (14‐Core) CPU, NVIDIA GeForce RTX 3080 Ti GPU, 64GB of memory. No additional hardware was needed to run the standalone Mk1C.

Using 10 R9.4.1 flow cells for sequencing, we used the integrated MinKNOW software (v.23.04.06) and Guppy basecalling (v.6.5.7) to conduct a total of 20 sequencing runs, each set up initially for 72‐h with runs manually ended by researcher upon significant pore depletion. During the second phase of sequencing, nuclease flushes were conducted after the completion of runs using the Flow Cell Wash kit (WSH‐004) following manufacturer's protocol, and additional aliquots of prepared library were loaded to perform additional sequencing runs. This modification allowed for up to four aliquots of 12 μl of DNA to be loaded on a given flow cell, significantly reducing the sequencing costs associated with the project. During all sequencing runs, Guppy FAST algorithm was used for initial real‐time basecalling.

### 2.2 Computational Methods

All bioinformatic analyses were performed on the Rutgers Amarel high performance computing (HPC) cluster. All scripts used for bioinformatic analyses are available at https://github.com/lhauff/Eulemur_genome_assembly.git.

#### 2.2.1 Rebasecalling and Trimming

To improve accuracy of reads originally generated in real‐time by Guppy FAST basecalling, *post hoc* basecalling was performed on all reads with SUP, a higher accuracy algorithmic model using Dorado (v.0.3.4). Briefly, this was achieved by first converting MinKNOW generated FAST5 files to POD5 format before using Dorado model dna_r9.4.1_e8_sup@v3.6 to reanalyze the files. Sequencing adaptors were then trimmed from the reads using Porechop (v.0.2.4; https://github.com/rrwick/Porechop). Sequencing summary statistics for the rebasecalled and trimmed reads were calculated with Nanoq (v.0.10.0; Steinig and Coin 2022).

#### 2.2.2 Initial Nuclear Genome Assembly

We next used a *de novo* assembler explicitly designed to accurately process long reads, Flye (v.2.9.1; Kolmogorov et al. 2019), to generate the initial *Eulemur rufifrons* assembly from the rebasecalled and trimmed reads. We selected *nano‐hq* as the read input type to account for the higher accuracy reads, and we estimated the genome size parameter as 3.0 Gb to accurately reflect a typical primate genome size.

#### 2.2.3 Polishing

We next performed extensive polishing of this draft assembly with Medaka (v.1.9.1, Oxford Nanopore Technologies, https://github.com/nanoporetech/medaka). To improve parallelism of this computationally intensive program, the Medaka program was split into the three discrete steps: *mini_align*, *medaka consensus*, and *medaka stitch*. Model r941_min_sup_g507 was specified during *medaka consensus*.

#### 2.2.4 Purging of Halpotigs

To remove allelic contigs, we then used Purge Haplotigs (v.1.1.2, Roach, Schmidt, and Borneman 2018), which uses read coverage to identify and remove likely haplotigs. Using our read coverage histogram, the following cutoffs were chosen to run the purging pipeline: low 5, medium 35, and high 75.

#### 2.2.5 Contamination Removal

To check the assembled contigs for contamination (bacteria, archaea, or virus), we then used Kraken2 (v.2.1.3; Wood, Lu, and Langmead 2019) with the standard database, which flagged definitively non‐primate reads for subsequent removal from the genome. Remaining contigs smaller than 3000 bp were then removed with *Seqkit seq* (v.2.5.1.; Shen et al. 2016) for additional assembly cleanup.

#### 2.2.6 Repeat Masking

To facilitate the speed and effectiveness of downstream annotation, we soft‐masked repetitive regions of the genome using RepeatMasker (v.4.1.5; Smit, Hubley, and Green 2013), with “Primate” indicated as species.

#### 2.2.7 Scaffolding

We then scaffolded the masked assembly to a *Lemur catta* reference genome (GCF_020740605.2_mLemCat1.pri, Palmada‐Flores et al. 2022), the most complete chromosome‐level Lemuridae reference genome available at the time of assembly, using RagTag (v.2.1.0; Alonge et al. 2022). To appropriately reflect our long‐read data, minimap2 parameters were set to “‐x map‐ont.” Contigs which mapped to the Y chromosome were subsequently removed as the specimen sequenced is female.

#### 2.2.8 Coverage and Quality Statistics

To initially assess assembly coverage and completeness, we first used QUAST (5.2.0; Mikheenko et al. 2018). This program provided information regarding genome statistics such as assembly length, average coverage depth, and NG50.

To further investigate the quality of our reference genome and to assess how it compares to other publicly available, annotated Lemuroidea genomes, we used BUSCO (v.5.7.0; Manni et al. 2021) to evaluate genome completeness based on expected gene content using Benchmarking Universal Single‐Copy Orthologs (BUSCO) databases. We specified the predictor to Miniprot (v.0.13; Li 2023), a protein‐to‐genome aligner and considered orthologues in vertebrate‐ (vertebrata_odb10) and primate‐ (primates_odb10) specific lineage databases. We did so for our *E. rufifrons* genome in addition to the other annotated Lemuroidea genomes ( *Lemur catta* , GCF_020740605.2_mLemCat1.pri, (Palmada‐Flores et al. 2022); *Propithecus coquereli* , GCF_000956105.1_Pcoq_1.0, (Guevara et al. 2021); and *Microcebus murinus* , GCF_000165445.2_Mmur_3.0, (Larsen et al. 2017)) for comparative purposes.

To generate the input files necessary to create a Blobtools2 directory for *Eulemur rufifrons* assembly visualization, we used BlobToolkit (v.4.3.5; Challis, Richards, and Rajan 2020) and the BUSCO generated results.

#### 2.2.9 Gene Annotation

To facilitate annotation, we generated pairwise alignment chains between our scaffolded *E. rufifrons* assembly and an annotated human reference genome (GRCh38.p14; Schneider et al. 2017) following the make_lastz_pipeline (https://github.com/hillerlab/make_lastz_chains) which uses LASTZ (v.1.04.22, https://github.com/lastz/lastz). We then used TOGA (v.1.1.5; Kirilenko et al. 2023) for homology‐based gene annotation. TOGA (Tool to infer Orthologs from Genome Alignments) projects orthologous loci for each reference transcript present in the provided reference genome, allowing for annotation and classification of the nearly 20,000 genes present in the gene model we used. Isoform and BED files (curated by the TOGA team from the human GENCODE V38 (Ensembl 104) gene annotation) necessary to obtain meaningful results were acquired within the TOGA GitHub (https://github.com/hillerlab/TOGA/TOGAInput/human_hg38) while the FASTA and GFF files for human genome were obtained directly from NCBI database (GCF_000001405.4).

#### 2.2.10 Mitochondrial Genome

To accurately assembly the mitochondrial genome, all rebasecalled reads were mapped to the *Lemur catta* mitogenome using minimap2 (v.2.14; Li 2021) and aligned reads were subsequently downsampled to 10,000 reads with SeqKit (v.2.5.1). With these subsampled reads, the mitogenome was assembled using Flye metagenome mode. The resulting circular contig with 273× coverage was recovered and polished using Medaka (v.1.9.2) as described previously. MITOS2 within the Galaxy web platform (Afgan et al. 2016) was consulted to confirm that the circular genome comprehensively contained mitochondrial genes. QUAST assembly statistics were calculated for the final mitogenome assembly as well.

## 3 Results

### 3.1 Sequencing and Rebasecalling

We extracted DNA from the skin and spleen of an *Eulemur rufifrons* specimen which had been opportunistically found within Ranomafana National Park and biobanked at the nearby Centre ValBio Research Station. High molecular weight extractions of the tissue sample yielded approximately 15 μg while DNA extractions from the spleen yielded over 25 μg, with the DNA remaining after sequencing efforts subsequently stored at −20°C at the research station. The sequencing efforts associated with the pilot stage resulted in lower sequencing success than our second attempt. The two distinct runs across two MinION flow cells (R9.4.1) loaded with high molecular weight (HMW) DNA library prepared with Field Sequencing Kit (Oxford Nanopore SQK‐LRK001) generated only 6.5 Gb of data and just over 180,000 reads. However, these efforts validated our field‐adapted protocol in rural Madagascar and provided training opportunities to Malagasy researchers on all associated genomic techniques.

Due to the modifications to our protocol as described above, the second stage of in‐country sequencing produced significantly improved sequencing data yields. During this stage, DNA was sequenced over 20 days using eight MinION flow cells (R9.4.1) across two portable sequencers (Mk1b and Mk1c). HMW DNA was used initially for library preparations with the Ligation Sequencing Kit (Oxford Nanopore SQK‐LSK109). However, upon depletion of that extraction and failure of the HMW extraction kit potentially due to field storage conditions, non‐HMW DNA from the spleen was used for remaining library preparations with the Ligation Kit as well. In total, over 36.4 million reads, comprising nearly 58.8 gigabases of DNA, were sequenced across all efforts. The second stage of sequencing allowed for additional training of Malagasy graduate students in associated genomic protocols.

Across both sequencing endeavors, Guppy (v6.5.7) FAST was used for real‐time basecalling, with reads being rebasecalled with the higher accuracy SUP algorithm using Dorado (0.3.4). After trimming the adapters with Porechop (v.0.2.4), all reads together had a N50 of 2611 (Table 1), which is expected due to significantly more short reads being generated by the use of non‐HMW DNA. Indeed, the average N50 across runs using only HMW DNA was 16.8 k.

**TABLE 1:** *E. rufifrons* nanopore sequencing summary statistics before and after rebasecalling with SUP algorithm using Dorado (v.0.3.4, model dna_r9.4.1_e8_sup@v3.6) and trimming with Prechop (v.0.2.4).

	Raw read estimates with HMW DNA	Raw read estimates with non‐HMW DNA	Read statistics after rebasecalling and trimming
Number of reads	7,068,390	29,529,670	36,553,682
Number of bases (bp)	19,539,340,000	31,403,000,000	57,550,868,813
N50 read length (bp)	16,800	1520	2611
Longest read (bp)	—	—	966,438
Mean read length (bp)	—	—	1574
Median read length (bp)	—	—	795
Mean read quality (QV)	—	—	7.33
Median read quality (QV)	—	—	13.58

*Note:* Raw read estimates were extracted from MinKNOW generated sequencing reports and post‐basecalling and trimming read statistics were generated with *Nanoq* with all reads across all sequencing efforts considered.

### 3.2 Genome Assembly and Curation

The initial assembly produced by Flye and polished with Medaka consisted of a total length of 2,205,502,242 bp in 17,373 contigs. The Purge Haplotigs pipeline identified and removed 5750 contigs (41,046,638 bp) as probable allelic contigs based on the following read coverage cutoffs: low 5, medium 35, and high 75. Kraken2 classified 97.55% of these remaining contigs (*N* = 11,338) to be putatively primate, while 3 contigs received basal classification of “cellular organisms,” 2.19% of the contigs (*N* = 257, 275,172 bp) remained unclassified, and 0.24% of these contigs (*N* = 28, 151,839 bp) were definitively classified as contamination and were subsequently removed. After soft‐masking, the assembly was then scaffolded to *Lemur catta* , the most complete chromosome‐level Lemuridae reference genome available at the time of assembly. This effort resulted in 3610 contigs (2,089,323,247 bp) successfully scaffolded to the 30 *Lemur catta* chromosomes serving as reference while 2951 contigs (58,980,565 bp) remained unplaced with 3580 gap sequences reported (11,615,937 bp) (Table S2). As the specimen sampled was female, any contigs that scaffolded to the Y‐chromosome in the *Lemur catta* reference genome were also considered erroneous and removed.

The mitochondrial genome assembly built by Flye and polished with Medaka produced a final singular circular contig of 17,108 bp with 1141× coverage and GC content of 39.08%, as determined by QUAST assembly statistics. The MITOS2 analysis annotated all expected mitochondrial genes.

**FIGURE 1:**
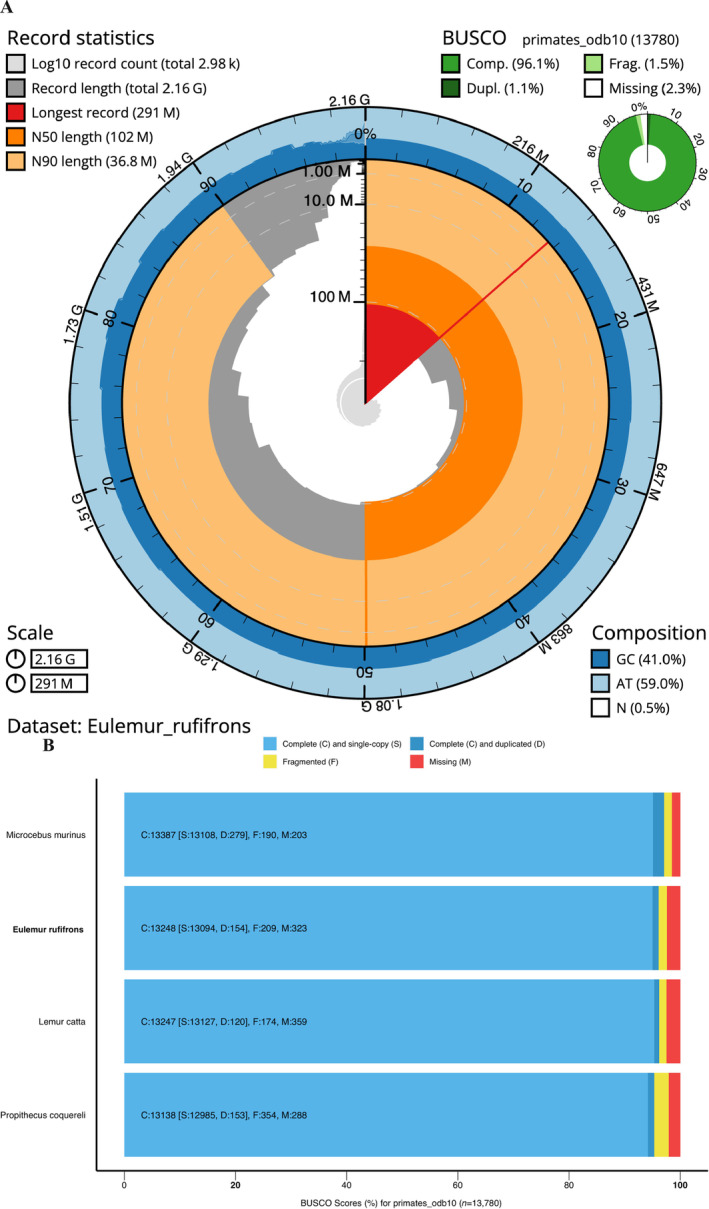
EulRuf_1.0 is a near‐complete and highly contiguous genome assembly for red‐fronted brown lemur ( *Eulemur rufifrons* ). (A) Final EulRuf_1.0 assembly statistics depicted with BlobtToolKit Snailplot showing total genome size of 2.16 Gb, an N50 of 102 Mb, and longest contig over 291 Mb, capturing all of chromosome 1. With an N90 over 36.8 Mb, the bulk of the assembly is represented by 23 contigs. (B) Lemuroidea annotated reference assembly completeness comparisons by locally searching primate‐specific genes (primates_odb10) with BUSCO (v.5.7.0). With a 96.13% BUSCO completeness score for primate‐specific genes, EulRuf_1.0 it is highly comparable to other high‐quality, Lemuroidae reference genomes.

QUAST assembly statistics of the nuclear genome indicated an N50 of 101,660,362, an L50 of 8, and an average coverage depth of 30×. The largest scaffold was 291,409,608 bp, which suggests that it has captured the entirety of chromosome 1 (286,674,946 in *Lemur catta* reference). GC content was also very similar to that of the *L. catta* reference genome: 40.95% to 41.36%, respectively. With an N90 over 36.8 Mb, the bulk of the assembly is represented by 23 contigs (Figure 1A). Based on these assembly quality statistics, our *Eulemur rufifrons* genome compares favorably to other publicly available Lemuroidea genomes (Table 2). After the addition of the polished mitogenome to the nuclear assembly, the resulting final assembly, EulRuf_1.0 (NCBI accession GCA_043251655.1), was of length 2,157,115,393 bp and consisted of 2981 scaffolds.

**TABLE 2:** Assembly statistics for Lemuroidea genomes considered for comparative purposes.

Assembly	Size (Gb)	Contigs/scaffolds/ chromosomes	Contig N50 (Mb)	Scaffold L50	Depth	Sample type
*Lemur catta* mLemCat1.pri	2.246	394/184/30	102.2	9	20.8×	spleen
*Microcebus murinus* Mmur_3.0	2.487	50,982/7677/34	198.2	10	221.6×	liver, kidney
*Propithecus coquereli* Pcoq_1.0	2.798	299,069/22,538/−	5.6	149	104.7×	kidney
***E. rufifrons*** **EulRuf_1.0**	**2.157**	**6562/2981/29**	**101.6**	**8**	**30×**	**Skin, spleen**

*Note:* Our *de novo* assembly for *Eulemur rufifrons* , generated with long‐read portable sequencing conducted entirely in the host country of Madagascar, is bolded.

To calculate comparable annotation completeness scores for all considered lemur genomes ( *Lemur catta* , GCF_020740605.2_mLemCat1.pri; *Propithecus coquereli* , GCF_000956105.1_Pcoq_1.0; and *Microcebus murinus* , GCF_000165445.2_Mmur_3.0), we assessed marker gene presence using BUSCO (v.5.7.0; Manni et al. 2021), considering the primate‐specific lineage dataset of 13,780 single‐copy orthologs (Figure 1B). The EulRuf_1.0 assembly achieved a 96.14% BUSCO completeness score for primate‐specific genes, making it highly comparable to other high‐quality, annotated Lemuridae reference genomes: *Microcebus murinus* (mmur_3.0) 97.15%, *Lemur catta* (mLemCat1.pri) 96.13%, *Propithecus coquereli* (Pcoq_1.0) 95.34%.

### 3.3 Genome Annotation

**FIGURE 2:**
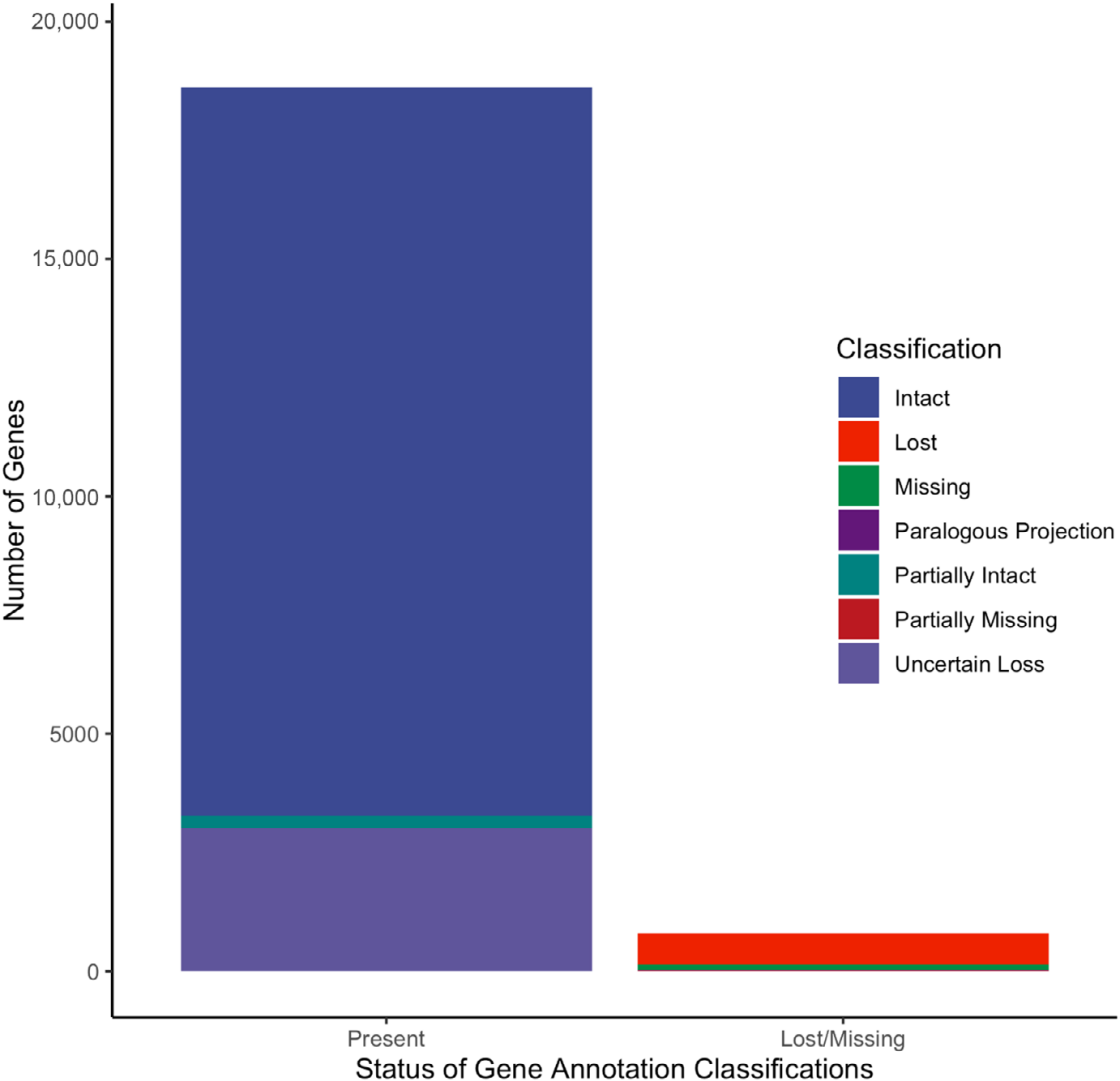
TOGA classifications for 19,414 human reference gene orthologs within *Eulemur rufifrons* . Genes considered to be present with informative annotations include 15,345 “Intact” genes (79.04%), 267 “Partially Intact” genes (1.38%), and 3008 genes with “Uncertain Loss” (transcripts with a single inactivating mutation in a single exon; 15.49%). Gene annotations that were deemed lost or missing include 647 genes “Lost” (3.33%), 124 genes “Missing” (0.64%), 5 “Paralogous Projections” (0.03%), and 18 genes “Partially Missing” (0.09%).

We used TOGA to annotate genes in the *E. rufifrons* genome, using the human genome (hg38; GCF_000001405.4) and corresponding isoform and BED files containing 39,664 transcripts of 19,464 genes as a reference (acquired from https://github.com/hillerlab/TOGA/TOGAInput/human_hg38). Additionally, as TOGA provides classifications regarding exon intactness, which relates to the likelihood that an annotated transcript in the query genome encoding a functional protein (Kirilenko et al. 2023), it can be used to further evaluate genome assembly quality. Of the 19,464 genes initially considered for annotation, TOGA was able to process 19,414, with 232 genes skipped, for various reasons including computational memory requirements. Considering these processed genes (Figure 2), TOGA reported 15,345 genes to be fully intact (79.04%); 267 genes to be partially intact (1.38%), which corresponds to greater or equal to 50% of transcripts considered to be found in coding region; and 647 genes (3.33%) to have at least two inactivating mutations in at least 2 exons, which TOGA considers “lost.” Genes classified as missing more than 50% of transcripts in the coding region due to assembly gaps or fragmentation totaled 124 (0.64%). Transcripts with a single inactivating mutation in a single exon are classified as “uncertain loss” and may be indicative of loss of an exon but not the entire gene (Kirilenko et al. 2023). TOGA classified 3008 genes (15.49%) as “uncertain loss” in the *E. rufifrons* genome while the remaining 5 genes were deemed “paralogous projections,” indicative of these genes being annotated but with inconclusive projections of transcripts.

With consideration that genes classified as “Intact,” “Partially Intact,” or “Uncertain Loss” by TOGA can all provide useful gene annotation evidence to other annotation tools (Kirilenko et al. 2023), such as EVidenceModeler (Haas et al. 2008), we considered the total of genes with informative annotations to 18,620, or 95.9%of the reference genes assessed. This value is comparable to other genome completeness metrics evaluated for this *de novo* assembly, providing further support that EulRuf_1.0 is a high‐quality genome.

## 4 Discussion

Here, we present EulRuf_1.0 (NCBI accession GCA_043251655.1), the first published high‐quality reference genome for the endangered red‐fronted brown lemur ( *Eulemur rufifrons* ) produced by reads generated entirely within the host country, Madagascar, on portable sequencers. With a contig N50 of 101.6 Mb and a 96.1 BUSCO completeness score for primate‐specific genes, this genome is highly comparable to other annotated Lemuroidea genomes currently available. Annotation efforts with TOGA resulted in 15,345 genes fully annotated, with partial transcripts annotated for an additional 3275 genes, bringing the total of informative gene annotations to 95.9% of the reference genes assessed. As the only annotated genome available at the time of publication for the speciose and widely distributed *Eulemur* genus, this reference genome will prove to be an important genomic resource for conservation efforts across Madagascar (Thorburn et al. 2023).

In addition to the success of our assembly efforts, these results highlight the potential of using portable sequencing technology to address both the biodiversity crisis and historical underrepresentation of researchers from the Global South in genomics research. With the ability to conduct whole‐genome sequencing projects at scale within host countries of high biodiversity, extraction of biological samples to sequencing centers concentrated in the Global North is no longer a genomics imperative. This enables more equitable international partnerships by facilitating enhanced training and participation in genomic work. Although some challenges were encountered while conducting sequencing in a remote region, such as expiration of consumables perhaps due to the tropical environment, our protocol proved to be adaptable and robust. This work joins the growing body of literature that showcase the technological advancements of portable, long‐read sequencing technologies and associated bioinformatic processes that enable the creation and curation of reference genomes for species of conservation concern (Flack et al. 2023; Pozo et al. 2024).

## Author Contributions

**Lindsey Hauff:** conceptualization (equal), data curation (lead), formal analysis (lead), funding acquisition (equal), investigation (lead), methodology (lead), visualization (lead), writing – original draft (lead), writing – review and editing (equal). **Noa Elosmie Rasoanaivo:** investigation (supporting), writing – review and editing (supporting). **Andriamahery Razafindrakoto:** investigation (supporting), writing – review and editing (supporting). **Hajanirina Ravelonjanahary:** investigation (supporting), writing – review and editing (supporting). **Patricia C. Wright:** funding acquisition (supporting), investigation (supporting), project administration (supporting), supervision (supporting), writing – review and editing (supporting). **Rindra Rakotoarivony:** investigation (supporting), supervision (supporting), writing – review and editing (supporting). **Christina M. Bergey:** conceptualization (equal), formal analysis (supporting), funding acquisition (equal), investigation (supporting), methodology (supporting), project administration (equal), supervision (lead), visualization (supporting), writing – original draft (supporting), writing – review and editing (equal).

## Conflicts of Interest

The authors declare no conflicts of interest.

### Open Research Badges

This article has earned an Open Data badge for making publicly available the digitally‐shareable data necessary to reproduce the reported results. The data is available at https://osf.io/hr67w.

## Supporting information


**Table S1.** Library preparation aliquots and associated sequencing run inputs.
**Table S2.** RagTag output report of scaffolding *de novo Eulemur rufifrons* assembly to *Lemur catta* reference genome.

## Data Availability

The sequencing data are available via NCBI BioProject (PRJNA1103329; https://www.ncbi.nlm.nih.gov/bioproject/?term=PRJNA1103329). This Whole Genome Sequencing assembly has been deposited at GenBank under the accession GCA_043251655. The version described in this paper is version GCA_043251655.1. The assembly, mitogenome annotation, and nuclear genome protein FASTA file are also available via Dryad (https://doi.org/10.5061/dryad.k3j9kd5gx). All code to reproduce the analyses can be found in a GitHub repository (https://github.com/lhauff/Eulemur_genome_assembly; DOI: 10.5281/zenodo.11225334).
