# Supplementary material for: De Novo Genome Assembly for an Endangered Lemur Using Portable Nanopore Sequencing in Rural Madagascar
Source: Ecol Evol. 2025 Jan 6;15(1):e70734. doi: 10.1002/ece3.70734 (PMC11705420; doi:10.1002/ece3.70734)
Supplement: Supplementary file 1 — Table S1. Library preparation aliquots and associated sequencing run inputs. Table S2. RagTag output report of scaffolding de novo Eulemur rufifrons assembly to Lemur catta reference genome. [file ECE3-15-e70734-s001.docx]

**Supplemental**

**Supplemental Table 1:** Library preparation aliquots and associated sequencing run inputs

| **Library Prep** | **Tissue Type of DNA extraction** | **Library Prep Kit** | **DNA input (µg)** | **Aliquots** |
| --- | --- | --- | --- | --- |
| 1 (pilot) | Skin | SQK-LRK001 | 1.5 | 1 |
| 2 (pilot) | Skin | SQK-LRK001 | .5 | 1 |
| 3 | Skin | SQK-LSK109 | 1.5 | 3 |
| 4 | Skin | SQK-LSK109 | 2.25 | 3 |
| 5 | Skin | SQK-LSK109 | 2.25 | 4 |
| 6 | Spleen | SQK-LSK109 | 3.5 | 4 |
| 7 | Spleen | SQK-LSK109 | 3.5 | 4 |

**Supplemental Table 2:** RagTag output report of scaffolding *de novo Eulemur rufifrons* assembly to *Lemur catta* reference genome

| **Placed sequences/bp** | **Unplaced sequences/bp** | **Gap sequences/bp** |
| --- | --- | --- |
| 3,610/2,089,323,247 | 2,951/58,980,565 | 3,580/11,615,037 |
